# Periodontal results of different therapeutic approaches (open vs. closed technique) and timing evaluation (< 2 year vs. > 2 year) of palatal impacted canines: a systematic review

**DOI:** 10.1186/s12903-021-01937-x

**Published:** 2021-11-10

**Authors:** Rosanna Guarnieri, Serena Bertoldo, Michele Cassetta, Federica Altieri, Camilla Grenga, Maurizio Vichi, Roberto Di Giorgio, Ersilia Barbato

**Affiliations:** 1 Department of Oral and Maxillofacial Sciences, School of Dentistry, “Sapienza” University of Rome, Via Caserta 6, 00161 Rome, Italy; 2 Department of Statistics, “Sapienza” University of Rome, Rome, Italy

**Keywords:** Palatal impacted canine, Surgical approach, Periodontal results, Therapeutic methods

## Abstract

**Background:**

This review evaluates, as a primary outcome, which surgical technique (open vs. closed) and which type of material used for the auxiliaries (elastic vs. metallic) were preferable in terms of periodontal results during the treatment of palatal-impacted canines. The timing of the evaluation of the results was also assessed as a secondary outcome.

**Methods:**

An electronic search of the literature up to March 2021 was performed on Pubmed, MEDLINE (via Pubmed), EMBASE (via Ovid), Cochrane Reviews and Cochrane Register of Controlled Trials (RCTs) (CENTRAL). The risk of bias evaluation was performed using version 2 of the Cochrane risk of bias tool (RoB 2) for RCTs and the ACROBAT NRSI tool of Cochrane for non-RCTs.

**Results:**

11 articles met the inclusion criteria. Only one RCT was assessed as having a low risk of bias and all the non-RCTs were assessed as having a serious risk of bias. This review revealed better periodontal results for the closed technique and metallic auxiliaries. In addition, it revealed that the timing of the evaluation of the results affects the periodontal results with better results obtained 2 years after the end of treatment.

**Conclusion:**

In the treatment of a palatal-impacted canine, the closed technique and metallic auxiliaries should be preferred in terms of better periodontal results. The timing of the evaluation of the results affects the periodontal results.

**Supplementary Information:**

The online version contains supplementary material available at 10.1186/s12903-021-01937-x.

## Background

The impaction of permanent maxillary canine affects 1–3% of the general population [1, 2]; this issue is twice as frequent in females as compared to males [3]; and is more frequently unilateral than bilateral [3, 4]. Palatal impaction occurs in 43–87% of impaction cases and is more common in Caucasian subjects than others [5–9].

Clinicians debate whether the open [10] or the closed [11] surgical approach during surgical-orthodontic treatment should be the treatment of choice for palatally-displaced canines.

Literature reviews which compare these two surgical techniques as the therapeutic approach to palatal-impacted canines have concluded that there is no difference between them regarding the periodontal outcomes [12, 13].

Concerning disimpaction treatment, there is no adequate literature on the different systems of force application and there is no quantitative analysis of these variables which can influence the results of the treated teeth, the contiguous teeth and periodontal health. During traditional fixed orthodontic treatments, the type of material used may have a different impact on the periodontal status, so this specific variable should be considered as a key factor in periodontal and dental results [12–14].

Furthermore, no previous systematic reviews reveal how the timing of the assessment can affect the results.

The aim of this study is to assess which therapeutic approach is preferable for palatal-impacted canines through a systematic review comparing the "open" and the "closed" surgical techniques and the system of forces application (elastic or metallic auxiliaries) (primary outcome). In addition, the influence of the timing of periodontal results evaluation was also assessed: results evaluated within 2 years from the end of treatment were compared with results assessed after 2 years from the end of treatment (secondary outcome).

The review was based on the PRISMA checklist [15].

## Methods

### Protocol and registration

Not available.

### Eligibility criteria

The following criteria were selected in this study.

#### Types of participants (P)

Orthodontic patients with unilateral, palatal impacted canines were included, with no age, race or type of malocclusion restrictions.

#### Types of interventions (I)

Three variables were analysed:

1. Surgical technique
2. Force application system
3. Timing of periodontal results evaluation.

#### Comparisons (C)

For the three aforementioned types of intervention:

1. Open technique versus Closed technique
2. Metallic auxiliaries (cantilever, ligatures, easy cuspid device, spring) versus elastic auxiliaries (ligatures, chains)
3. Periodontal results evaluation: < 2 years after the end of treatment vs. > 2 years after the end of treatment.

#### Outcomes (O)

The following periodontal indices were considered: PD: Probing depth, PI: Plaque Index, REC: Recession, KT: Keratinized Tissue, CL: Crown length, CAL: Clinical Attachment Level.

#### Included study types (S)

Randomized Controlled Trials (RCTs), Quasi-RCT (Q-RCTs), Controlled Clinical Trials (CCTs), unclear Non-Randomized Studies (uNRS), Prospective and Retrospective Studies were included in this study. Case reports were excluded.

Eligibility criteria comprised of: only articles published in English after 1990; description of periodontal results and description of the system of forces application included.

Exclusion criteria were the period of publication, non-English language, studies that did not report adequately periodontal results or the description of both surgical approach and forces application system.

### Search strategy

The studies were identified by bibliographic research of electronic databases, examining the bibliography of the articles.

The bibliographic research was carried out on Pubmed, MEDLINE (via Pubmed), EMBASE (via Ovid), Cochrane Reviews and Cochrane Register of Controlled Trials (CENTRAL).

The research was performed up to March 2021.

The search was carried out using a combination of controlled vocabulary and free text terms and Boolean operators.

The free text keywords used for PubMed are shown in Table 1.

**Table 1:** Keywords used for pubmed search

	Keywords	Items found
1	Palatally impacted canine	360
2	Palatal canine impaction	433
3	Palatally displaced canine	192
4	Canine impaction surgical orthodontic treatment	370
5	Canine impaction surgical orthodontic treatment effect	34
6	Palatal impacted canine treatment	304
7	Palatally displaced canine treatment	104
8	Palatal canine impaction AND treatment	302
9	Palatal displaced canine AND treatment	109
10	Palatal canine AND treatment AND side effect	56
11	Palatally impacted canine AND treatment AND side effect	8
12	Palatally impacted canine AND treatment AND periodontal status	22
13	Palatal impacted canine AND treatment AND surgical orthodontic	130
14	Palatal impacted canine AND open technique	20
15	Post-treatment AND palatal impacted canine	14
16	Adverse effect AND treatment AND palatal impacted canine	5
17	Side effect AND treatment AND palatal impacted canine	9

### Study selection

The selection of the studies was carried out independently by two of the Authors (S.B. and R.G.). The degree of accuracy and agreement between the two authors was assessed using Cohen's kappa coefficient (κ). Any disagreement was resolved by a third Author (F.A.). Potentially adequate studies were initially identified through the evaluation of the title. Abstracts of the non-excluded studies were read and studies that did not match the eligibility criteria were eliminated. At the next stage, full texts were examined and items that did not match the eligibility criteria were excluded.

### Data collection process

The data were collected into an excel file and then reviewed by two of the Authors (S.B. and R.G.). Any disagreements were resolved through comparison with a third Author (F.A.).

### Data item

Information regarding the studies is shown in Table 2.

**Table 2:** Information about data items

Authors	Type of study	No. patient	Age	Sex	Surgery approach	Type of anchorage	Force application system	Statistical analysis	Timing of evaluation of the results	Periodontal results	Receiving funding
Parkin et al. [18]	RCT	62. (group 1: 33 canines; group 2: 29 canines)	Group 1: 14.2 yrs; group 2: 14 yrs	Group 1: 11 M and 22 F. group 2: 8 M and 21 F	Group 1: OT group 2: CT	Dental: fixed appliance	Twin-wire technique or an elastic chain	t test, chi-square test, McNemar test, Wilcoxon signed rank test	3 months after fixed appliances removal	CAL, REC and alveolar bone level was statistically relevant. CL was not statistically relevant	Yes
Smailiene et al. [19]	Q-RCT	43. (group 1: 22; group 2: 21) (control group: contralateral teeth)	Group 1: 18.6 ± 3.45 yrs; group 2: 19.7 ± 4.37 yrs	8 M and 35 F	Group 1: OT and free eruption. group 2: CT	Dental: fixed appliance with rectangular stabilization archwire	Group 2: ballista loop on the additional stainless steel archwire	Kolmogorov–Smirnov test, Student’s t-test, non-parametric Mann–Whitney U-test, Student’s (t) paired test, non-parametric Wilcoxon, Pearson’s test, Spearman’s test, chi-square test	4.19 ± 1.44 months (3–6 months) after fixed appliance removal	PD and Bone support were statistically relevant. REC and KT did not differ significantly	NS
Hansson and Rindler [27]	uNRS	42. (control group: contralateral teeth)	14–42 yrs	15 M and 27F	11 Canines: CT; 31 Canines: OT	Dental: lingual arch with occlusal stay on the adjacent premolar	Spring attached either to the first molar band or to the lingual arch	Student-t test. Wilcoxon test, Dahlberg's formula	1 to 18 yrs (mean 12.3 yrs) post-treatment	PI mesial and palatal to the canine was higher compared to the control group. PD mesial to the canine was higher compared to the control group. GI: no difference between treated and control group. Bone level distal to the canine was lower compared to the control group	Yes
Szarmach et al. [21]	Prospective	24. (Control group: contralateral teeth)	18.4 ± 3.66 yrs	5 M and 19 F	–	Dental: fixed appliance with rectangular steel arch	Accessory steel arch with a “ballista” loop	Student t-test, non-parametric Wilcoxon test and Pearson correlation coefficient	After canine alignment	PD, CAL: statistically significant. PI: statistically insignificant	NS
Zafarmand and Gholami [20]	prospective	20. (control group: contralateral teeth)	16.7 ± 1.9 yrs	10 M and 10 F	OT (modified window technique)	Dental: fixed appliance (archwire)	Elastic thread	Mann–Whitney U test	6 months after therapy	BOP was found in 8 patients, CL was greater in the study group than in the control group, KT were lower in the study group, CAL were lower in the study group. Range of bone level were not statistically significant	NS
Mummolo et al. [10]	prospective	19. (9 palatal and 10 buccal)	19.44 ± 2.4 yrs (palatal group); 18.5 ± 1.96 yrs (buccal group)	5 M and 5 F (buccal) e 4 M e 5 F (palatal)	OT (buccal group: apically repositioned full-thickness mucoperiosteal flap. Palatal group: operculectomy)	Dental: fixed appliance	Elastic thread	Mann–Whitney U test, test P, Post Hoc	12 months after the end of orthodontic treatment	PD was higher in treated groups than in their respective control groups. KT was generally lower in both treatment groups than in their respective control groups	No
Crescini et al. [26]	Retrospective	15. (8 palatal and 7 buccal). (Control group: contralateral teeth)	14 yrs e 8 months	4 M and 11F	CT	Dental: fixed appliance	Elastic traction	Student- t test	After an average period of 39 months	CAL, REC not statistically relevant. PI e BOP increased compared to the control group. KT was lower compared to the control group	NS
Zasciurinskiene et al. [25]	retrospective	32. (control group: contralateral teeth)	18.2 ± 5.1 yrs	10 M and 22F	CT	Dental: palatal arch at the start and fixed appliance later	Ligation chain	Nonparametric Kruskal–Wallis test, Mann–Whitney U-test	3 months after removal of the fixed appliances	Mean PD at the mesiopalatal point on the treated canine was greater than in contralateral canines. REC had non-significant values	NS
Caprioglio et al. [23]	Retrospective	33. (control group: contralateral teeth)	12.4–24.1 yrs	9 M and 24 F	CT	Dental: fixed appliance + Trans-palatal arch	Easy Cuspid device	Student t -test	4.6 years after the end of the active treatment phase	PD differences were not statistically significant	NS
Evren et al. [24]	Retrospective	30. (group 1: 15 Palatal; group 2: 15 Buccal) (Control group: contralateral teeth)	11.43 ± 1.5 yrs		Group 1: CT	-	-	T-test, Wilcox test, Mann.Whitney U-test	3.82 ± 1.54 years after the orthodontic treatment	Group 1 had a higher PD and a lower bone level compared to the control group. Variation of PI, GBI, CAL loss and REC were no statistically significant	NS
Bollero et al. [22]	Retrospective	28. (group 1:14 buccal; group 2:14 palatal) (Control group: contralateral teeth)	13yrs and 5 months ± 1 yr and 4 months	Group 1: 7 M and 7F, group 2: 6 M and 8F	Group 2: CT	Dental: fixed appliance + quad-helix canine system	Elastic tie	T-test, Wilcox test, Mann–Whitney U-test	After a mean period of 2 yrs 4 months ± 1 yr 1 month following the removal of the orthodontic appliances	Group 2: PD was greater mesio-palatally compared to the control group. No statistical difference in the PI, BOP, REC, KT between group 2 and the control group	NS

*RCT* randomized controlled trial, *Q-RCT* quasi-randomized controlled trial, *uNRS* unclear non randomized study, *yr(s)* year(s), *M* male, *F* female, *OT* open technique, *CT* closed technique, *PD* probing depth, *PI* Plaque Index, *REC* recession, *KT* keratinized tissue, *CL* crown length, *CAL* clinical attachment level, *NS* non-specified

### Risk of bias assessment

The risk of bias was assessed independently by two Authors (S.B. and R.G.). Any disagreement was resolved by two other Authors (C.G. and F.A.). The study quality of randomized and quasi-randomized studies was determined using version 2 of the Cochrane risk of bias tool (RoB 2) to the domains shown in Table 3 [16]. The result can be 'Low' or 'High' risk of bias or can express 'Some concerns'.

**Table 3:** Risk of bias for randomized trials

Authors	Risk of bias arising from the randomization process	Risk of bias due to deviations from the intended interventions	Missing outcome data	Risk of bias in measurement of the outcome	Risk of bias in selection of the reported result	Overall risk of bias
Parkin et al. [18]	Low	Low	Low	Low	Low	Low
Smailiene et al. [19]	High	Low	Low	Low	Low	High

The non-randomized trials were assessed by ACROBAT NRSI tool of Cochrane to the domains shown in Table 4 [17].

**Table 4:** Risk of bias for non randomized trials

Authors	Confounding bias	Selection of participants bias	Measurement of interventions bias	Departures from intended interventions bias	Missing data bias	Measurements of outcomes bias	Selection of the reported results Bias	overall bias
Hansson and Rindler [27]	Serious	Low	Serious	Low	Moderate	Moderate	Low	Serious
Mummolo et al. [10]	Serious	Low	Moderate	Low	Low	Moderate	Low	Moderate
Bollero et al. [22]	Serious	Serious	Serious	Low	Serious	Moderate	Low	Serious
Caprioglio et al. [23]	Serious	Low	Moderate	Low	Moderate	Moderate	Low	Moderate
Evren et al. [24]	Serious	Moderate	Serious	Low	Serious	Serious	Low	Serious
Zasciurinskiene et al. [25]	Serious	Moderate	Moderate	Low	Low	Low	Low	Moderate
Crescini et al. [26]	Serious	Low	Serious	Low	Low	Serious	Low	Serious
Zafarmand and Gholami [20]	Serious	Serious	Moderate	Low	Moderate	Moderate	Low	Moderate
Szarmach et al. [21]	Serious	Moderate	Moderate	Low	Serious	Serious	Low	Serious

Possible results for each domain and for the overall results were: 'low', 'moderate', 'serious', 'critical' risk of bias and 'no information'.

### Effect measures and synthesis methods

Mean values and standard deviations (DS) were used in order to express the estimate of effect. Where standard deviations were missing, they were calculated and then all the values were reported in tables shown in the *additional files* (*Af*). Specifically, Additional file 1: Table S1.*Af* reports periodontal indices according to the surgical technique, Additional file 1: Table S2.*Af* reports periodontal indices according to the system of force application, Additional file 1: Table S3.*Af* reports periodontal indices regarding the surgical technique, classified on the results of the evaluation timing, Additional file 1: Table S4.*Af* reports periodontal indices regarding the system of force application, classified on the results evaluation timing.

## Results

### Study selection

A total of 3217 studies were identified through the database search. 1651 of these studies were eliminated because the title was not related to the research that was being carried out. After the duplicates were deleted, 297 items remained. Abstracts were read and 213 articles were excluded because they did not meet the inclusion criteria: 53 articles were deleted because they were published before 1990, 93 articles were excluded because of the study type, 41 articles were excluded due to the therapeutic approach type, 26 were excluded because it was not specified whether the results were for palatal or buccal impacted canines.

The full text of the remaining 84 articles were examined. 69 were excluded because they did not match the eligibility criteria: 4 of them were excluded because the full text was not in English, 39 studies did not report periodontal results or the force application system. 30 articles did not report the force application system.

**Fig. 1:**
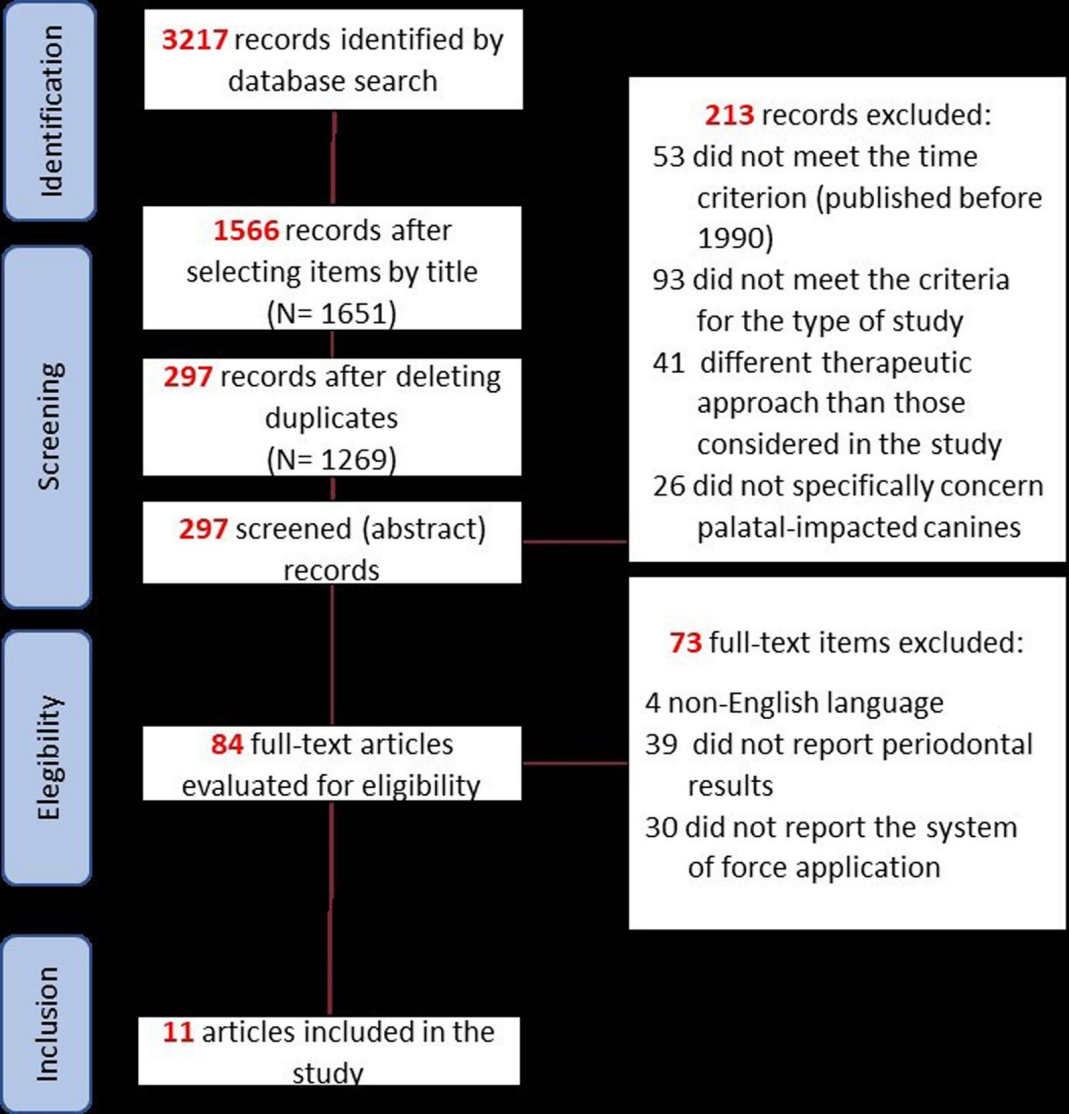
Study selection flowchart

Following the application of the eligibility criteria, 11 articles remained. The selection procedure is represented in Fig. 1.

Cohen’s kappa coefficient (κ) was 0,857 showing an excellent strength of agreement amongst the Authors.

### Study characteristics

Of the 11 items, one study was an RCT [18], one was a Q-RCT [19], three were prospective studies [10, 20, 21], five were retrospective studies [22–26] and there was one unclear Non-Randomized Study (uNRS) [27]. A total of 364 subjects were analyzed across all the studies.

Parkin’s et al. [18] study compares subjects treated with the open and closed technique. The force application system was not adequately explained so it was not considered. Smailiene's et al. [19] study compares open and closed techniques to each other and against contralaterals, but in this study the closed technique-treated group was excluded from the quantitative and qualitative results assessment because it was followed by a spontaneous eruption which is not the subject of this study. The force application system used was metallic. Mummolo’s et al. [10] study compares subjects treated with the open technique and elastic force application system to the contralaterals. In Zafarmand and Gholami’s [20] study subjects treated with open technique and an elastic force application system are compared to the contralaterals. In Szarmach’s et al. [21] study subjects treated with open technique and metal force application system with contralaterals were compared. Bollero’s et al. [22] study compares subjects treated with closed technique and elastic forces application system to the contralaterals. Caprioglio’s et al. [23] study compares impacted canines treated with closed technique and force application system using metal ligatures with the untreated contralaterals. Evren’s et al. [24] study compares canines treated with closed technique and contralaterals not specifying the type of force application system used for disimpaction. In Zasciurinkiene’s et al. [25] study subjects treated with closed technique and using a metallic auxiliary to apply the force system were compared to the contralaterals. Crescini et al. [26] compares subjects treated with closed technique and with an elastic force application system with the contralaterals. Hansson and Rindler’s [27] study compares canines treated with open and closed technique with the contralaterals.

Further information about study characteristics is collated in Table 2.

### Risk of bias

The results for randomized and quasi-randomized studies are shown in Table 3.

Regarding the sequence generation and the allocation concealment Smailiene’s et al. [19] study was at high risk of bias because it described an inappropriate method of random sequence generation. It is impossible to blind participants or the dentist to the surgical procedure, but assuming that the surgeon was equally experienced at both techniques, it unlikely that this would introduce a high degree of bias due to deviations from the intended interventions. For this reason, the studies were judged to be at low risk of bias for this domain. Since missing outcome data were balanced in numbers across intervention groups, with similar reasons for missing data across groups, the studies were judged to be at low risk of bias for this domain. RCTs studies were judged to be at low risk of bias in measurement of the outcome because the statement about primary or secondary outcomes was satisfactorily clear. Regarding risk of bias in selection of the reported result, all studies were considered at low risk.

Regarding the non-randomized trials, the results are shown in Table 4.

For each separate outcome different confounders were considered for the assessment of risk of bias such as the initial position and the angle of canine (orientation, inclination and depth of impaction), oral hygiene, initial periodontal condition, age and gender. All the non-RCTs were assessed as having a serious risk of bias.

### Synthesis of results

Parkin’s et al. [18] study reports no difference between canines exposed through an open versus a closed surgical technique, but it reported a statistical difference in certain periodontal indices when comparing operated and contralateral (unoperated) canine. It concludes that there is no difference in periodontal health for impacted canine treated with either the open or closed technique. In Smailiene's et al. [19] study it was reported that there were no significant differences with respect to periodontal pocket depth. However, compared with contralateral normally erupted canines, impacted canines showed a significant bone loss at a specific site of the canine. Mummolo’s et al. [10] concluded that the periodontal status of palatal impacted canine was not affected by surgical-orthodontic treatment with an open technique. In Zafarmand and Gholami’s [20] study they did not report a significant difference between the periodontal status of the 2 groups except for the level of alveolar bone that was significantly lower in the surgically-exposed group. Szarmach's et al. [21] showed an overall inferior periodontal result in the experimental group compared to the control group, especially in specific periodontal sites. In Bollero's et al. [22] study, palatally impacted canines exhibited a significantly greater PD on the mesiolingual site when compared to their contralaterals while the other periodontal results were not significant. Caprioglio's et al. [23] study reports that the use of a closed-flap surgical technique in association with a codified orthodontic traction system revealed no significant clinical differences regarding periodontal indices compared to the control group. Evren's et al. [24] study, reports that palatally impacted canines treated with the closed technique had worse periodontal indices values compared to their contralaterals. Zasciurinkiene's et al. [25] showed that a significant increase in pocket depth was found at specific sites of the canine after surgical-orthodontic treatment, but the periodontal conditions were considered clinically acceptable. The Crescini et al. [26] study reported that no significant differences in the periodontal indices were observed between test and control teeth at the follow-up examination. Hansson and Rindler’s [27] study results showed good periodontal status with slight differences between treated and untreated teeth.

In this study, the Authors used the mean values of the periodontal indices to compare them. Due to the heterogeneity of the available data, it was impossible to perform an appropriate meta-analysis, therefore a qualitative evaluation was performed based on the values shown in the additional files.

Regarding the surgical approach it was possible to note a higher PD value in the open technique than in the closed one, a lower KT value in the open technique than in the closed one, a higher CAL value in the closed technique than in the open one, a higher REC value (based on Crown Length difference) in the open technique than in the closed.

According to the system of force application, the following outcomes were found: a higher PD using elastic auxiliaries than metallic ones, a smaller amount of KT using elastic auxiliaries than using metal, a higher PI using elastic auxiliaries than using metal auxiliaries, a higher REC using metal auxiliaries than using elastic ones, a higher CAL using elastic auxiliaries than using metal auxiliaries.

Based on the timing of the results evaluation, the following results were found: a higher PD for evaluations made within 2 years of the end of the orthodontic treatment when compared to those made after 2 years, a lower amount of KT for evaluations made within 2 years than for those made after 2 years, a higher REC for evaluations made within 2 years than for those made after 2 years, a higher CALs for evaluations made within 2 years than those evaluated after 2 years.

## Discussion

### Summary of evidence

This study included a total of 280 treated palatal impacted maxillary permanent canines. Regarding the comparison between open and closed technique, the qualitative assessment of the periodontal outcomes showed overall better results using a closed technique; this is in accordance with some results of Wisth et al. [28], who reported better results for closed technique in terms of PD and CAL, but unfortunately this study was retrospective with a high risk of bias. The better results of the closed technique could be due to the following factors: better preservation of the CEJ, better soft tissue healing, less plaque accumulation and better post-operative comfort [29, 30].

Diversely, the results found are in contrast with previous studies where no differences were found when comparing these two surgical techniques [12, 18, 19, 31]; furthermore, even if there were differences in periodontal indices, these were not significant.

There is a lack of literature regarding the system of force application. In fact, in the included articles there are no comparative studies regarding the use of either elastic or metallic auxiliaries. Therefore, the analyses done in this study are based on comparisons made between different studies. This study compares elastic and metallic auxiliaries and shows how the use of metallic auxiliaries gives better periodontal results, independently from the surgical technique used. It is probably associated with the lower quantity of plaque accumulation using metallic auxiliaries as the lower PI mean value of this study confirms, in accordance with other studies [32, 33].

Regarding how the timing of results’ evaluation affects results, the present study shows better results after a minimum of a two-year period after the end of treatment, independent of the type of surgical technique used. The retrospective study of Crescini et al. [26] analyzed the results at the baseline (at the end of the treatment) and after a three-year period and they found that there are no relevant differences in periodontal status except for the average PD value which decreased over time. However, this study has a high risk of bias; furthermore it evaluated both palatal and buccal impacted canines (multiple comparisons that increase test sensitivity) and it is a retrospective study which has lower scientific evidence compared to the systematic review [34]. The fact that periodontal indices are better after a 2-year period following the end of the treatment could be associated with the physiological regenerative capacity of the tissue, as Crescini et al. [26] mentioned in their study, which also demonstrated that most of the PD increased values found at the baseline, were actually due to pseudo-pockets.

### Limitations

This systematic review has the following limitations:

- There was no differential assessment based on the age of the participants
- There is high heterogeneity of the results
- The samples size is quite limited in certain studies

In addition, an important limitation is that in the closed technique, the type and the extension of the flap was not accurately evaluated, and an assessment of the instrumentation used for the open technique was not carried out.

Another limit is that the auxiliaries used for the force application system were assessed based only on the type of material (elastic or metallic) and not on a specific type of auxiliary which can affect the periodontal and dental results.

## Conclusion

1. Periodontal results, excluding the CAL results, were found to be better with the closed technique rather than with open technique.
2. Metallic-auxiliaries offer a better post-treatment periodontal condition.
3. Results evaluation timing influences the outcomes. Specifically, outcomes evaluated at least 2 years after the end of the overall orthodontic treatment are better than the outcomes evaluated up to 2 years post-treatment.

Finally, more high-quality studies should be performed in the future to enable stronger evidence. In the future it would be also interesting to investigate how skeletal anchorage, associated with both open and closed technique, affect periodontal indices of palatal impacted canines.

## Supplementary Information

**Additional file 1.** Tables reporting periodontal indices.

## Data Availability

The datasets used and/or analyzed during the current study are available from the corresponding author upon reasonable request.

## Abbreviations

PD: Probing depth
PI: Plaque Index
REC: Recession
KT: Keratinized tissue
CL: Crown length
CAL: Clinical attachment level
RCTs: Randomized controlled trials
Q-RCTs: Quasi-RCT
CCTs: Controlled clinical trials
uNRS: Unclear non-randomized studies
NS: Non-specified
Af: Additional files
