# Supplementary material for: Periodontal results of different therapeutic approaches (open vs. closed technique) and timing evaluation (< 2 year vs. > 2 year) of palatal impacted canines: a systematic review
Source: BMC Oral Health. 2021 Nov 10;21:574. doi: 10.1186/s12903-021-01937-x (PMC8579516; doi:10.1186/s12903-021-01937-x)
Supplement: Supplementary file 1 — Additional file 1. Tables reporting periodontal indices. [file 12903_2021_1937_MOESM1_ESM.docx]

**Additional file 1**

*Table 1. Af.* Periodontal Indices mean values and SDs, classified according to the surgical technique. PD (Probing depth), KT (Keratinized Tissue), CL (Crown length), CAL (Clinical Attachment Level), SD (Standard Deviation)

| **Author** | **n patients** | **Timing of evaluation of result** | **Type of comparisons** | **PD impacted canine** | **DS** | **KT impacted canine** | **DS** | **CAL impacted canine** | **DS** | **CL impacted canine** | **DS** |
| --- | --- | --- | --- | --- | --- | --- | --- | --- | --- | --- | --- |
| Bollero 2017 | 14 | 2 years 4 months ± 1 year 1 month (mean value) after the end of orthodontic treatment | palatal canine vs controlateral canine: **closed technique**, dental anchorage, elastic tie | 2.20 | 0.61 | 4.33 | 0.82 |  |  |  |  |
| Caprioglio 2012 | 33 | 4.6 years after the end of orthodontic treatment | palatal canine vs controlateral canine. **closed technique**, dental anchorage, easy cuspid device | 1.21 | 0.52 |  |  |  |  |  |  |
| Crescini 1994 | 8 | 39 months (3.25 years) (mean value) after the end of orthodontic treatment | palatal canine vs controlateral canine. **closed technique**, dental anchorage, elastic traction | 2.14 | 0.44 | 5.13 | 1.37 |  |  |  |  |
| Evren 2014 | 15 | 3.82 ± 1.54 years after the end of orthodontic treatment | palatal canine vs controlateral: **closed technique** | 1.63 | 0.33 |  |  | 3.20 | 0.86 | 10.27 | 0.46 |
| Hannson 1998 | 42 | 1 to 18 years (mean 12.3 years) after the end of orthodontic treatment | palatal canine vs controlateral canine. **closed technique**, dental anchorage, spring | 1.74 | 0.76 |  |  |  |  |  |  |
| Smailiene 2013 | 21 | 4.19 ± 1.44 months (3–6 months) after fixed appliance removal | closed technique vs controlateral: **closed technique**, dental anchorage, ballista loop on the additional archwire | closed 2.41 | 0.98 | 5.11 | 1.05 |  |  |  |  |
| Zasciurinskiene 2008 | 32. | 3 months after fixed appliance removal | palatal canine vs controlateral: **closed technique**, dental anchorage, ligation chain | 2.53 | 1.04 |  |  |  |  |  |  |
| Mummolo 2018 | 9 | 12 months after the end of orthodontic treatment | impacted palatal canine vs controlateral: **open technique**, dental anchorage | 2.33 | 0.50 | 3.22 | 0.75 |  |  |  |  |
| Zafarmand 2009 | 20. | 6 months after the end of orthodontic treatment | palatal impacted canine vs controlateral: **open technique**, dental anchorage, elastic thread | 2.325 | 0.9 | 4.5 | 1.4 | 2.6 | 0.7 | 9.9 | 1.6 |

*Table 2. Af.* Periodontal Indices mean values and SDs, classified according to the system of force application. PD (Probing depth), PI (Plaque Index), REC (Recession), KT (Keratinized Tissue), CAL (Clinical Attachment Level), SD (Standard Deviation)

| **Author** | **n patients** | **Timing of evaluation of result** | **Type of comparisons** | **PD impacted canine** | **SD** | **KT impacted canine** | **SD** | **PI impacted canine** | **SD** | **REC impacted canine** | **SD** | **CAL impacted canine** | **SD** |
| --- | --- | --- | --- | --- | --- | --- | --- | --- | --- | --- | --- | --- | --- |
| Bollero 2017 | 14 | 2 years 4 months ± 1 year 1 month (mean value) after the end of orthodontic treatment | palatal canine vs controlateral canine: closed technique, dental anchorage, **elastic** tie | 2.20 | 0.61 | 4.33 | 0.82 | 0.67 | 0.52 | 0.00 | 0.00 |  |  |
| Crescini 1994 | 8 | 39 months (3.25 years) (mean value) after the end of orthodontic treatment | palatal canine vs controlateral canine. Closed technique, dental anchorage, **elastic** traction | 2.14 | 0.44 | 5.13 | 1.37 |  |  |  |  |  |  |
| Mummolo 2018 | 9 | 12 months after the end of orthodontic treatment | impacted palatal canine vs controlateral: open technique, dental anchorage, **elastic** thread | 2.33 | 0.50 | 3.22 | 0.75 |  |  |  |  |  |  |
| Zafarmand 2009 | 20 | 6 months after the end of orthodontic treatment | palatal impacted canine vs controlateral: open technique, dental anchorage, **elastic** thread | 2.325 | 0.9 | 4.5 | 1.4 |  |  |  |  | 2.6 | 0.7 |
| Zasciurinskiene 2008 | 32 | 3 months after fixed appliance removal | palatal canine vs controlateral: closed technique, dental anchorage, **ligation chain (metallic)** | 2.53 | 1.04 |  |  |  |  | 0.97 | 0.11 |  |  |
| Caprioglio 2012 | 33 | 4.6 years after the end of orthodontic treatment | palatal canine vs controlateral canine. Closed technique, dental anchorage, **easy cuspid** **device (metallic)** | 1.21 | 0.52 |  |  |  |  |  |  |  |  |
| Hansson 1998 | 42. | 1 to 18 years (mean 12.3 years) after the end of orthodontic treatment | palatal canine vs controlateral canine. Closed technique, dental anchorage, **spring (metallic)** | 1.74 | 0.76 |  |  | 0.40 | 0.56 |  |  |  |  |
| Smailiene 2013 | 21 | 4.19 ± 1.44 months (3–6 months) after fixed appliance removal | closed technique vs controlateral: closed technique, dental anchorage, **ballista loop (metallic)** on the additional archwire | closed 2.41 | 0.98 | 5.11 | 1.05 |  |  | 0.03 | 0.15 |  |  |
| Szarmach 2006 | 24 | after canine alignment | palatal impacted canine vs controlateral: dental anchorage and **ballista loop (metallic)** on the accessory arch | 2.02 | 1.02 |  |  |  |  |  |  | 1.44 | 0.98 |

*Table 3.Af*: Periodontal Indices mean values and SDs regarding the surgical technique, classified on the results evaluation timing. PD (Probing depth), KT (Keratinized Tissue), REC (Recession), CL (Crown length), CAL (Clinical Attachment Level), SD (Standard Deviation)

| **Author** | **n patients** | **Timing of evaluation of the results** | **Type of comparisons** | **PD impacted canine** | **SD** | **KT impacted canine** | **SD** | **REC impacted canine** | **SD** | **CAL impacted canine** | **SD** | **CL impacted canine** | **SD** |
| --- | --- | --- | --- | --- | --- | --- | --- | --- | --- | --- | --- | --- | --- |
| Zasciurinskiene 2008 | 32 | 3 months after fixed appliance removal | palatal canine vs controlateral: **closed technique**, dental anchorage, ligation chain | 2.53 | 1.04 |  |  | 0.97 | 0.11 |  |  |  |  |
| Zafarmand 2009 | 20 | 6 months after the end of orthodontic treatment | palatal impacted canine vs controlateral: **open technique**, dental anchorage, elasti thread | 2.325 | 0.9 | 4.5 | 1.4 |  |  | 2.6 | 0.7 | 9.9 | 1.6 |
| Mummolo 2018 | 9 | 12 months after the end of orthodontic treatment | impacted palatal canine vs controlateral: **open technique**, dental anchorage | 2.33 | 0.50 | 3.22 | 0.75 |  |  |  |  |  |  |
| Bollero 2017 | 14 | 2 years 4 months ± 1 year 1 month (mean value) after the end of orthodontic treatment | palatal canine vs controlateral canine: **closed technique**, dental anchorage, elastic tie | 2.20 | 0.61 | 4.33 | 0.82 | 0.00 | 0 |  |  |  |  |
| Crescini 1994 | 8 | 39 months (3.25 years) (mean value) after the end of orthodontic treatment | palatal canine vs controlateral canine. **closed technique**, dental anchorage, elastic traction | 2.14 | 0.44 | 5.13 | 1.37 |  |  |  |  |  |  |
| Evren 2014 | 15 | 3.82 ± 1.54 years after the end of orthodontic treatment | palatal canine vs controlateral: **closed technique** | 1.63 | 0.33 |  |  |  |  | 3.20 | 0.86 | 10.27 | 0.46 |
| Smailiene 2013 | 21 | 4.19 ± 1.44 months (3–6 months) after fixed appliance removal | closed technique vs controlateral: **closed technique**, dental anchorage, ballista loop on the additional archwire | closed 2.41 | 0.98 | 5.11 | 1.05 | 0.03 | 0.15 |  |  |  |  |
| Caprioglio 2012 | 33 | 4.6 years after the end of orthodontic treatment | palatal canine vs controlateral canine. **closed technique**, dental anchorage, easy cuspid device | 1.21 | 0.52 |  |  |  |  |  |  |  |  |
| Hannson 1998 | 42 | 1 to 18 years (mean 12.3 years) after the end of orthodontic treatment | palatal canine vs controlateral canine. **closed technique**, dental anchorage, spring | 1.74 | 0.76 |  |  |  |  |  |  |  |  |

*Table 4. Af.* Periodontal Indices mean values and SDs regarding the system of force application, classified based on the results evaluation timing. PD (Probing depth), KT (Keratinized Tissue), REC (Recession), SD (Standard Deviation)

| **Author** | **n patients** | **Timing of evaluation of the results** | **Type of comparisons** | **PD impacted canine** | **SD** | **KT impacted canine** | **SD** | **REC impacted canine** | **SD** |
| --- | --- | --- | --- | --- | --- | --- | --- | --- | --- |
| Szarmach 2006 | 24 | after canine alignment | palatal impacted canine vs controlateral: dental anchorage and **ballista loop** on the accessory arch | 2.02 | 1,02 |  |  |  |  |
| Zasciurinskiene 2008 | 32 | 3 months after fixed appliance removal | palatal canine vs controlateral: closed technique, dental anchorage, **ligation chain (metallic)** | 2.53 | 1,04 |  |  | 0.97 | 0.11 |
| Zafarmand 2009 | 20 | 6 months after the end of orthodontic treatment | palatal impacted canine vs controlateral: open technique, dental anchorage, **elastic** thread | 2.325 | 0.9 | 4.5 | 1.4 |  |  |
| Mummolo 2018 | 9 | 12 months after the end of orthodontic treatment | impacted palatal canine vs controlateral: open technique, dental anchorage, **elastic** thread | 2.33 | 0.5 | 3.22 | 0.75 |  |  |
| Bollero 2017 | 14 | 2 years 4 months ± 1 year 1 month (mean value) after the end of orthodontic treatment | palatal canine vs controlateral canine: closed technique, dental anchorage, **elastic** tie | 2.20 | 0.61 | 4.33 | 0.82 | 0.00 | 0 |
| Crescini 1994 | 8 | 39 months (3.25 years) (mean value) after the end of orthodontic treatment | palatal canine vs controlateral canine. Closed technique, dental anchorage, **elastic** traction | 2.14 | 0.44 | 5.13 | 1.37 |  |  |
| Smailiene 2013 | 21 | 4.19 ± 1.44 months (3–6 months) after fixed appliance removal | closed technique vs controlateral: closed technique, dental anchorage, **ballista loop (metallic)** on the additional archwire | closed 2.41 | 0.98 | 5.11 | 1.05 | 0.03 | 0.15 |
| Caprioglio 2012 | 33 | 4.6 years after the end of orthodontic treatment | palatal canine vs controlateral canine. Closed technique,dental anchorage, **easy cuspid** **device (metallic)** | 1.21 | 0.52 |  |  |  |  |
| Hansson 1998 | 42 | 1 to 18 years (mean 12.3 years) after the end of orthodontic treatment | palatal canine vs controlateral canine. Closed technique, dental anchorage, **spring (metallic)** | 1.74 | 0.76 |  |  |  |  |
